# Intracortical Microelectrode Array Unit Yield under Chronic Conditions: A Comparative Evaluation

**DOI:** 10.3390/mi12080972

**Published:** 2021-08-17

**Authors:** Joshua O. Usoro, Brandon S. Sturgill, Kate C. Musselman, Jeffrey R. Capadona, Joseph J. Pancrazio

**Affiliations:** 1 Department of Bioengineering, The University of Texas at Dallas, Richardson, TX 75080, USA; joshua.usoro@utdallas.edu (J.O.U.); brandon.sturgill@utdallas.edu (B.S.S.); katecmusselman@gmail.com (K.C.M.); 2 Department of Biomedical Engineering, Case Western Reserve University, Cleveland, OH 44106, USA; jrc35@case.edu; 3 Advanced Platform Technology Center, L. Stokes Cleveland VA Medical Center, Rehabilitation Research and Development, Cleveland, OH 44106, USA

**Keywords:** neural interface, microelectrode array, intracortical, chronic, active electrode yield

## Abstract

While microelectrode arrays (MEAs) offer the promise of elucidating functional neural circuitry and serve as the basis for a cortical neuroprosthesis, the challenge of designing and demonstrating chronically reliable technology remains. Numerous studies report “chronic” data but the actual time spans and performance measures corresponding to the experimental work vary. In this study, we reviewed the experimental durations that constitute chronic studies across a range of MEA types and animal species to gain an understanding of the widespread variability in reported study duration. For rodents, which are the most commonly used animal model in chronic studies, we examined active electrode yield (AEY) for different array types as a means to contextualize the study duration variance, as well as investigate and interpret the performance of custom devices in comparison to conventional MEAs. We observed wide-spread variance within species for the chronic implantation period and an AEY that decayed linearly in rodent models that implanted commercially-available devices. These observations provide a benchmark for comparing the performance of new technologies and highlight the need for consistency in chronic MEA studies. Additionally, to fully derive performance under chronic conditions, the duration of abiotic failure modes, biological processes induced by indwelling probes, and intended application of the device are key determinants.

## 1. Introduction

Neural interfaces have garnered tremendous interest in recent years due to their ability to record from and stimulate neuronal populations. These devices have provided an increased understanding of neuronal network dynamics underlying behavior and enabled a variety of clinical applications ranging from controlling text and cursors on a screen [1,2,3] to restoring lost motor and sensory functionality [4,5,6,7,8,9]. Neural interfaces for the central nervous system range from completely non-invasive (e.g. electroencephalography scalp electrodes), to epi/subdural electrocorticography arrays, to invasive intracortical microelectrode arrays (MEAs). While these devices all have tradeoffs between resolution of neural activity and invasiveness, intracortical MEAs have attracted significant attention due to their ability to interface with individual neurons and neural ensembles, which when coupled with recent advancements in computational capabilities, allows for a relatively high level of specificity and volitional control [10].

Intracortical MEAs are characterized by electrodes with geometric surface areas that are typically smaller than 10,000 µm^2^ [11], that record and/or stimulate from within the cortex. Historically, these MEAs have been classified into three types of devices: microwire arrays, fabricated planar arrays, and machined arrays. Microwires are comprised of a core conducting wire generally constructed from tungsten, stainless steel, iridium, or platinum, and an insulator that typically consists of glass, parylene-C, polyimide, or Teflon. The insulation is commonly removed/exposed at the tip to create the recording site. 

Planar arrays are historically fabricated from a rigid silicon substrate using photolithographic techniques in which layers of conductive and nonconductive materials are deposited in thin films to build up a planar structure. Conductive traces and electrodes are often made from metals such as gold or iridium and are insulated using polymer coatings similar to those used in microwires. 

Machined arrays use a dicing, etching, and glass reflow process to mill a block of silicon into a bed of penetrating needle-shaped shanks. Electrode material (often platinum or iridium) is then deposited on the shanks, followed by an insulator that is etched away at the tips to expose the recording sites, similar to microwires. 

Regardless of the type, each of the MEAs suffers from reliability issues over long periods of implantation. While there are both biotic and abiotic factors that influence reliability [12,13,14], the neural interfaces field is exploring a wide range of approaches spanning novel constituent materials and coatings [15,16,17,18] to ultrasmall dimensions [19,20,21] to pharmacological interventions [22,23,24] to achieve chronic reliability. With rapid advancement in MEA technology however, the question arises as to how to best contextualize and compare both the performance of novel devices to other custom-built or conventional arrays, as well as the duration for demonstrating this performance.

In this study, we performed a comprehensive evaluation of studies published within the past 35 years that conducted experiments using intracortical MEAs where authors claimed either “chronic” or “long-term” experimentation. We then compiled and analyzed the data by correlating the corresponding timepoints within studies to a variety of metrics to gain a better understanding of the criteria involved in determining chronic performance. Furthermore, for studies involving rodents, the most widely used animal model for chronic experimentation, we evaluated the utility of using active electrode yield as a means to contextualize and compare the performance of novel and conventional MEAs. What emerges is a consensus- and biologically-based rationale for chronic study duration, as well as a performance metric guided by a decay rate that informs the evaluation of prior work and the design of future MEA studies for fundamental and preclinical research. 

## 2. Materials and Methods

### 2.1. Search Strategy

A systematic search was conducted to collect a representative sample of the chronic intracortical MEA literature. The following search terms were used to identify articles in Google Scholar, PubMed, and Web of Science: chronic, long-term, intracortical implantation, microelectrode array, chronic MEA, brain interface, neural interface, and cortical electrode array. Additional manual searches were performed using the references of articles that were identified for inclusion in the study.

### 2.2. Inclusion/Exclusion Criteria

This study only included peer-reviewed articles that reported implanting a microelectrode array into the cortex of the brain for a “chronic” or “long-term” duration. A microelectrode array was defined as a having an electrode geometric surface area of less than 10,000 µm^2^ [11], thereby excluding many studies involving deep brain stimulating electrodes. Studies were excluded if they reported a “semi-chronic” timepoint or if they alluded to a chronic duration but did not provide an actual number of days/weeks over which the study was conducted. Articles reporting the use of electrocorticography arrays were also excluded as these devices typically lie on surface of the brain and do not penetrate the cortex. 

### 2.3. Selection and Sorting of Studies 

After the initial search, titles and abstracts were evaluated to determine relevance to the study. Articles that passed the initial assessment and appeared likely to meet the inclusion criteria were then analyzed using the full text. Emphasis was placed on preclinical studies in order to better assess trends within academic and institutional research environments. Once it was confirmed that the article met the inclusion criteria, pertinent data was extracted and sorted by animal model, device used, and year published. The “Animal Model” grouping was broadly split into 5 categories: (1) Mouse, (2) Rat, (3) Small Animal (bird (finch/budgerigar), cat (domestic), guinea pig (domestic), pig (domestic), rabbit (domestic)), (4) Non-human Primate, and (5) Human. The “Device Used” grouping was divided into 4 categories: (1) Microwire, (2) NeuroNexus (planar arrays), (3) Blackrock (machined arrays), and (4) Custom, i.e., any study that described in-house fabrication or modification of commercially-available devices. This was done to intrinsically separate the commercial devices by fabrication methods, dimensions, and applications, as these could have a large effect on chronic duration. The “Year Published” grouping was conducted in 5-year increments. The chronic time point was recorded as the researcher’s intended experimental design duration. If none was explicitly stated, the time point at which the majority of data collection ended. For example, there were multiple studies in which a researcher continued to collect data somewhat indefinitely from one remaining animal to determine how long physiological signals could be detected. If multiple time points were reported (e.g., histological endpoints), the longest duration was recorded.

### 2.4. Active Electrode Yield

For studies involving the use of rodents, active electrode yield (AEY), defined as the percentage of electrodes that recorded one or more identifiable units, was extracted at the earliest and latest timepoints that the data were reported. AEY was chosen as the representative performance metric due to its consistency in reporting, and similarity in acquisition and calculation across studies, as opposed to measures such as signal-to-noise ratio, electrochemical properties, and histological outcomes, which can vary drastically between studies and devices [24,25,26,27,28,29]. As an example, consider the utility of SNR across multiple studies. Kozai et al. (2017) defined SNR as PP/(2*σ) where PP is the average peak-to-peak amplitude of an identified single unit and σ is the standard deviation or, equivalently, the root mean square of the noise [30]. In contrast, Mols et al. (2017) defined SNR as PP/(6*σ) whereas others may have quantified SNR in their analyses but not offered a definition in the text (Luan et al., 2017) [31,32]. Curve fitting was conducted on AEY as a function of post-implantation duration to determine decay rates associated with potential decreases in device performance. A curve was considered “fit” if the Chi-Square tolerance was below 1 × 10^−9^ and the adjusted R^2^ value exceeded 0.5.

## 3. Results

The initial search yielded approximately 300 articles, of which 158 articles (162 data points) were ultimately chosen for inclusion in this survey. A few human studies were included to provide a more complete overview of intracortical MEA use, but the vast majority of selected articles and the ensuing analysis focused on preclinical models originating in North America. To examine trends in how the term “chronic” was used, articles were grouped based on the animal model and device reported in the study, as well as the year the study was published. 

**Figure 1:**
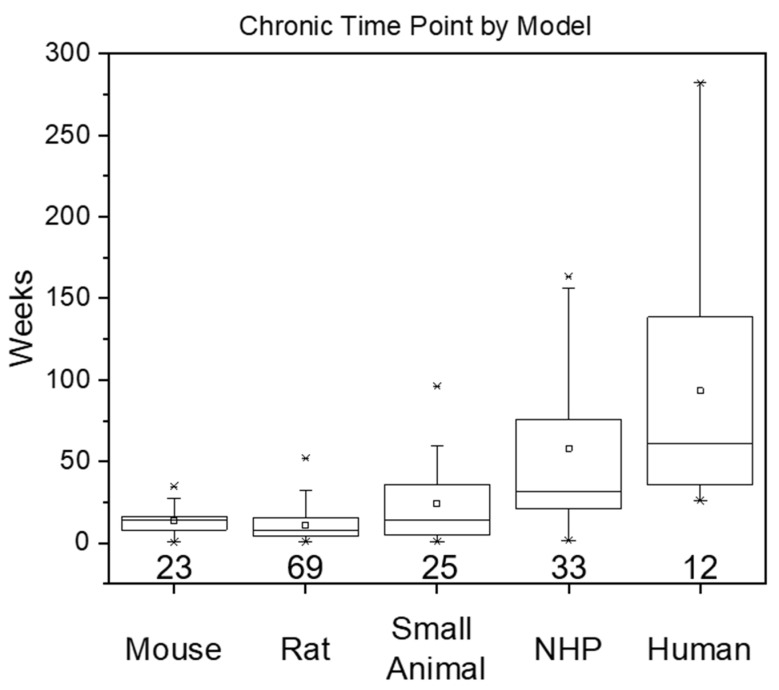
Chronic durations based on animal species. The box is determined by the 25th and 75th percentiles while the whiskers indicate 1.5 times the interquartile range. The minimum and maximum are represented by asterisks while the horizontal line and small square within the box indicate the median and mean, respectively. The numbers below each box and whisker plot reflect the number of studies contributing to the plot.

The animal model group was broken down by size and prevalence in research, resulting in separate categories for mouse and rat, as well a “small animal group” for less commonly used species. For studies that reported using multiple animal models, each species was included as a distinct entry for data collection purposes, but did not affect the overall total number of articles. A breakdown of the groupings can be seen in Figure 1 and Table 1.

The rat model was the most common of the animal models with a relatively even distribution among the mouse, small animal, and non-human primate group. The mouse and rat models exhibited similar means and medians, whereas the remaining groups displayed increasing chronic durations that scaled to the expected lifespan of the animal model. The variation within each group was quite large, with standard deviations ranging from 64-96% of the mean and medians that substantially differed from the means. This widespread variation highlights the lack of established standards in the field regarding the definition or use of the term “chronic” in this experimental context. Furthermore, the mouse, rat, and small animal models all included studies that reported chronic durations that were ≤1 week, while the non-human primate group included a chronic time point that was ≤2 weeks, despite mounting evidence that the dynamic phase of the neuroinflammatory response due to probe implantation may last for several weeks and the neuroinflammatory response due to the MEA may continue for 8–16 weeks [12,14,65,77,174,175,176]. Interestingly, the increase in animal model size and complexity also mapped to an increased average life expectancy [177], suggesting that the demonstration and expectation of device longevity and performance may be influenced by animal model selection (Supplementary Figure S1). 

**Figure 2:**
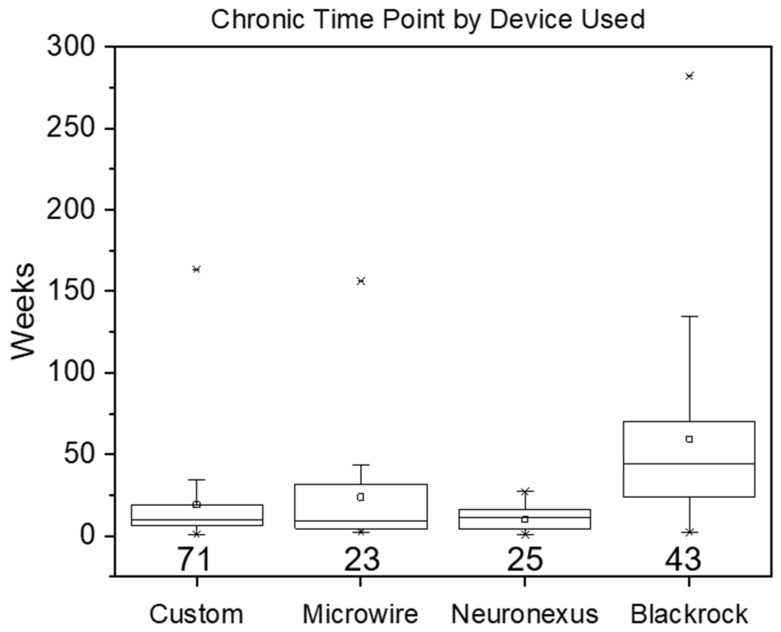
Chronic durations based on implanted device. The box is determined by the 25th and 75th percentiles while the whiskers indicate 1.5 times the interquartile range. The minimum and maximum are represented by asterisks while the horizontal line and small square within the box indicate the median and mean respectively. The numbers below each box and whisker plot reflect the number of studies contributing to the plot.

The use of the term chronic was also explored as a function of the type of implant used: Microwire, Neuronexus, Blackrock, or Custom. Figure 2 shows that the majority of studies used custom-made devices in their experiments, with all groups reporting high levels of variation similar to Figure 1.

It is worth noting that the means of each device group appear to correlate with different animal models. The Neuronexus, Microwire, and Blackrock averages reflect the rat, small animal, and non-human primate/human models, respectively. This is not surprising for the latter, given that most of the work performed in non-human primates and humans utilize the Blackrock array. When examining the medians however, the same trend is not observed (Supplemntary Table S1). The Custom, Microwire, and Neuronexus groups all reflect medians similar to that of the rat/mouse model. Additionally, the median of the Custom device group falls on the shorter end of the spectrum, on par with Microwires which were traditionally used in acute experiments. This may be because custom devices are typically examined in a mouse/rat model which provides a low cost and well-studied means of testing novel arrays. To this end, we also asked how the device used may influence the definition of a chronic duration in the mouse and rat model (Supplementary Tables S2 and S3). Supplmentary Tables S2 and S3 confirm that custom arrays were in fact the most widely used devices in the mouse and rat models. In the rat model, the means and medians were comparable across Custom, Microwire, and Neuronexus arrays, indicating that custom devices were not held to a different standard of chronic duration when compared to some commercially-available devices. Blackrock devices in rats did exhibit a longer duration, however. Additionally, there were no substantial differences observed in the mean or median chronic duration when comparing the devices used in the mouse model.

Extending this analysis to the other animal models, a longer duration was observed for small animal studies utilizing the Blackrock array as compared to the other array types (Supplementary Table S4). The mean chronic duration for non-human primate studies were consistent, but the median did increase from Custom, to Microwire, to Blackrock arrays, and for the human model there is insufficent data due to the blackrock array being almost exclusievely used (Supplementary Tables S5 and S6). As seen in Supplementary Table S7 no trends were observed for chronic duration as a function of the study publication year; furthermore, Supplementary Tables S8–S12 have data for chronic duration by publication year for each animal model.

Additionally, It is possible that the country where the research was conducted may influence durations of studies based on local social, ethical or legal considerations. We investigated whether there were differences between chronic time points in different geographical regions (Supplementary Tables S13–S16). We found that there was still widespread variability within each of the geographical regions we reviewed (North America (0.4–282 weeks), Europe (0.7–30 weeks), Asia (4–98.6 weeks), Other (1–36 weeks)) with comparable medians and/or means. The overwhelming majority of studies constituting this review originated from North America (132/158) indicating that the trends observed are unlikely to be influenced by country of origin.

In an effort to gain insight into array performance across device types and studies over reported chronic duration, we extracted information related to recording performance, i.e., the ability of the array to measure physiologically relevant signals. AEY is generally reported as a percentage of electrodes that recorded one or more identifiable units and is valuable as a recording metric because of its consistency between animal models (underlying electrophysiology remains the same) and studies (calculated as a simple percentage), as opposed to the numerous formulas used to calculate signal-to-noise ratio, as well as electrochemical measurements that vary widely based on electrode dimension, roughness, and material composition. Because AEY is not as widely reported in larger animal experiments, and due to the high number of, and average duration similarities between, mouse and rat studies, our analysis focused on a combined rodent model. 

**Figure 3:**
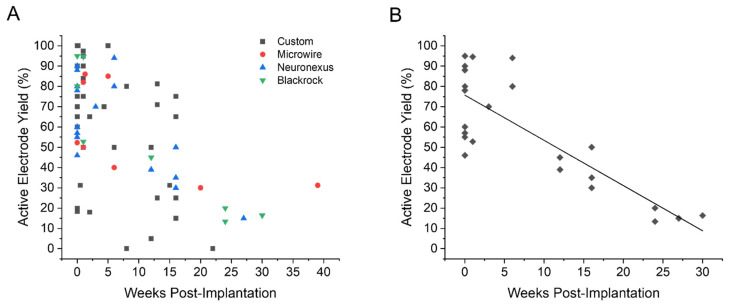
Active electrode yield over time. (**A**) A scatterplot of initial and final active electrode yield percentages for Custom (black square), Microwire (red circle), Neuronexus (blue triangle), and Blackrock (green inverted triangle) as reported by the original studies. (**B**) Curve fitting of a scatterplot consisting of commercially-fabricated devices (Neuronexus and Blackrock). The curve resulted in an initial AEY value of ~75% and slope of –2.2 percent/week with adjusted R^2^ and Pearson’s R values of 0.67 and –0.83 respectively. Data used to create Figure 3 was extrapolated from the references as follows: Custom [16,30,32,51,52,55,61,62,71,84,88,92], Microwire [58,63,81,82], Neuronexus [24,25,26,27,44,68,91], Blackrock [29,98,99,103].

Figure 3A shows reported AEY measurements across different device types. Microwire, Neuronexus, and Blackrock arrays all exhibited a general decline in AEY over time, with yields at or below 30% after 16 weeks post-implantation. Custom arrays, however, did not exhibit any discernible trend over time, with studies reporting AEY between 15–75% at Week 16, highlighting the widespread variance in the performance of custom arrays. The distribution of Custom AEY appeared almost bimodal, with most yields falling between either 15–25% or 65–75%. 

We recognize that novel devices comprising the “custom” group may not have fully mature fabrication processes. As a result, custom devices may have a lower fabrication yield characterized by fewer functional microelectrode sites or entire devices that were non-functional. Often, impedance measurements were used to qualify devices at an individual channel basis [30,92]. For example, Luan et al. (2017) reported a fabrication yield for connected microelectrode channels of 83% for the ultraflexible intracortical probes. Likewise, Guan et al. (2019) reported that processing steps to produce neurotassel technology resulted in a device yield of >80%. In each case, studies excluded non-functional microelectrode sites and, in some cases, entire devices when evidence indicated that the custom probes exhibited physical defects [32,46].

The stark difference in performance at similar timepoints is likely, in part, due to the various fabrication techniques and material composition used across studies, emphasizing the need for methodology to aid in the direct comparison of novel arrays. To this end, we investigated the use of curve fitting as a means to calculate the decay rate of AEY. Because our goal was to evaluate a standardization method for comparison of novel devices, we focused our curve fitting analysis on Neuronexus and Blackrock arrays (n = 24), the current standards for commercially-available MEA technology, and the most widely used and directly comparable devices across studies. Several fitting models were tested on the commercially-available AEY distribution including single and two-phase exponential decays, with the linear model providing the best fit. The linear model resulted in an adjusted R^2^ value of 0.67 and a Pearson’s R of −0.83, indicating a strongly negative correlation between AEY and time (as expected) with a model fit that far exceeded chance (Figure 3B). The linear fit produced an initial AEY value of approximately 75% and slope of −2.2 percent/week, suggesting that these commercially-available devices can be expected to exhibit a relatively high level of performance early on in a study, but may experience a decline of approximately 50% in performance over the course of 16 weeks.

## 4. Discussion

In this study, we report how researchers define the term “chronic” in intracortical microelectrode array applications. To our knowledge, this is the first systematic review on the use and interpretation of chronic duration in this field to date. By analyzing literature across various animal models and devices, this study highlights how variable the use of “chronic” or “long-term” is. For example, we see studies from North America tend to have longer time points than the rest of the globe (Table S14). This may be due to differences in regulations affecting study duration or animal models used in each region. Although the mean time point for North America may be skewed to longer durations by a few studies, the median is similar to the European studies used in this review. Together this accounts for most of the studies observed. Additionally, the median time point appears to be converging over the last decade. However, there is a large range of timepoints noted as chronic across studies. It is noteworthy that the majority of the studies, but not all, have durations that are at least 8 weeks in duration consistent with a biologically relevant timepoint. This reaffirms the need for a common interpretation so comparisons between studies for evaluating the long-term performance of this technology are possible. Moreover, our evaluation of the decay rate of AEY, an electrophysiological performance measure common to many MEA studies, may be useful in helping to contextualize the long-term performance of novel arrays by serving as a generalizable standard of comparison. 

### 4.1. Intended Application and Abiotic Considerations

The primary function of intracortical MEAs is to serve as a gateway into the nervous system, interfacing with the body to enhance our understanding of the underlying physiology and enable applications in motor and sensory functional improvements. In chronic experimentation, it is therefore of paramount importance to first characterize the long-term use of these devices based on their intended application and the ability of the device to perform as expected throughout the duration of anticipated use. Given the ever-increasing number of ways in which this technology could be utilized, intended application in this context can be broadly generalized and divided into two categories: basic science and task performance. For studies aiming to answer questions regarding the fundamental science underlying physiology (e.g., foreign body response, plasticity, molecular pathways, etc.), the chronic duration of the experiment will largely be determined by the time course of the physiologic occurrence in question. Furthermore, these types of studies are typically carried out in a rodent model due to its low cost and well-studied nature, so chronic duration may also be influenced by the animal model selection as well. For studies that aim to evaluate the use of MEAs to aid in performing specific tasks however (e.g., control of neuroprostheses, moving a computer cursor, or functional electrical stimulation), one could argue that the rehabilitative nature of these applications necessitate chronic durations that ideally last the lifetime of the patient. Given the current limitations of this technology though [12,13,14,178], it is unrealistic to expect MEAs to maintain functionality for this long. What may be a more useful approach in determining chronic durations is to partially base them on the limitations themselves. MEA failure modes can be categorized as either biotic, abiotic, or a combination of both. This study does not go into detail about abiotic failure modes (see [13,39,82,179]), but given the myriad materials, geometries, fabrication methods, and surgical techniques involved in implementing MEAs, it becomes increasingly difficult to pinpoint a chronic duration based on this metric alone, signifying that a range of durations may be necessary to account for the vast differences between devices.

For research in which the primary purpose is to demonstrate long-term device functionality (electrophysiological or electrochemical), it may be prudent to include a performance metric component alongside the experimental duration rationale. Similar to the AEY decay rate evaluated in this study (Figure 3), using comparable device- or animal-specific decay curves may provide a means by which to interpret the success of new technology, irrespective of material composition, geometries, or fabrication methods. Furthermore, utilizing these performance-based metrics in combination with experimental duration rationale may also aid in the interpretation of the performance of novel technology when compared to commercially-available devices. 

### 4.2. Biological Considerations

The biological neuroinflammatory response to the implanted device is believed to be a major source of intracortical microelectrode failure, and should be considered, in part, in the definition of the time course of deployment within a living system. The biological response to intracortical microelectrodes is well-characterized, but many of the relationships between device performance and the dynamic and changing environment adjacent to the implanted microelectrodes remains unknown. In brief, it is well-known that there are at least two phases of the foreign body response, acute and chronic. The acute phase of inflammation is defined by the exudation of fluids and plasma proteins, as well as the migration of leukocytes to the site of injury [180]. In general, acute inflammation typically only lasts from minutes to days in response to most medical device implantation. However, with respect to intracortical microelectrodes, the blood-brain barrier adds a unique dynamic. Several researchers have demonstrated that the blood-brain barrier remains leaky to both serum proteins and leukocytes as long as the intracortical microelectrodes remain implanted, suggesting that acute inflammation never completely subsides [85,181,182,183], despite the onset of a chronic inflammatory response and glial scar formation [184]. Due to the dual existence of both acute and chronic inflammatory states around the intracortical microelectrodes, and the regular micromotion of the microelectrode relative to the adjacent tissue [185,186,187], there is a continued foreign body response that persists through the indwelling period, although relatively static and consistent in their processes [188], meaning there is no sudden dynamic or drastic shift in their behavior barring any externally-triggered stimulus. The point at which the biological response reaches what is essentially a steady-state, should therefore be the minimum time point at which the evaluation of a device is considered chronic. Numerous articles reporting analysis in a rodent model found strong evidence that reduced blood-brain barrier integrity and neuroinflammation that were acutely caused by vascular disruption, transitioned to a sub-chronic phase at 6 weeks that was characterized by endogenous tissue events [77,85,188,189,190]. Furthermore, these studies suggested that this response reaches a steady state at 12–16 weeks post implantation but varies largely based on the design and materials composition of the individual microelectrode [191].

## 5. Conclusions

Based on the literature survey, there is a large variation in the duration of studies that investigate the chronic performance of implanted MEAs. A focused evaluation of rodent studies that utilized commercially-available devices revealed that there is a consistent decay rate in AEY that may serve as a benchmark for comparing emerging technology. It is important to recognize that AEY does not capture the consistency of individual units over time. For this reason, AEY is necessary but perhaps insufficient to predict reliability for brain computer interface implementations that require unit consistency. Expanding on prior work that suggests that a minimum 12–16 week indwelling period may be sufficient for the assessment of chronic performance, the addition of a suitable performance metric such as AEY decay rate might aid in the contextualization and interpretation of the long-term functionality of this technology. 

## Figures and Tables

**Table 1:** Chronic time point statistics based on model.

Metric (in Weeks)	Mouse	Rat	Small Animal	Non-Human Primate	Human
Number of Studies	23	69	25	33	12
Mean	13.5	10.8	25.1	58.0	93.3
Median	12.0	8.00	14.3	31.9	61.0
Range	0.429–34.7	1.00–52.0	0.700–96.0	1.43–163	26.0–282
Standard Deviation	8.70	9.40	23.3	53.0	77.3
References	[24,25,31,32,33,34,35,36,37,38,39,40,41,42,43,44,45,46,47,48,49,50]	[16,22,26,27,28,29,33,38,48,51,52,53,54,55,56,57,58,59,60,61,62,63,64,65,66,67,68,69,70,71,72,73,74,75,76,77,78,79,80,81,82,83,84,85,86,87,88,89,90,91,92,93,94,95,96,97,98,99,100,101,102,103,104,105,106,107,108,109,110]	[111,112,113,114,115,116,117,118,119,120,121,122,123,124,125,126,127,128,129,130,131,132,133,134,135]	[3,133,136,137,138,139,140,141,142,143,144,145,146,147,148,149,150,151,152,153,154,155,156,157,158,159,160,161,162,163,164]	[1,4,7,165,166,167,168,169,170,171,172,173]

## Data Availability

Not applicable.

## Supplementary Materials

The following are available online at https://www.mdpi.com/article/10.3390/mi12080972/s1, Figure S1: Chronic durations based on life expectancy, Table S1: Chronic time point statistics based on device used, Table S2: Chronic time point statistics based on device used in a mouse model, Table S3: Chronic time point statistics based on device used in a rat model, Table S4: Chronic time point statistics based on device used in a small animal model, Table S5: Chronic time point statistics based on device used in a non-human primate model, Table S6: Chronic time point statistics based on device used in a human model, Table S7: Chronic time point statistics based on publication year, Table S8: Chronic time point statistics based on publication year in a mouse model, Table S9: Chronic time point statistics based on publication year in a rat model, Table S10: Chronic time point statistics based on publication year in a small animal model Table S11: Chronic time point statistics based on publication year in a non-human primate model, Table S12: Chronic time point statistics based on publication year in a human model, Table S13: Chronic time point statistics based on geographical region, Table S14: Chronic time point statistics based on animal model in North America, Table S15: Chronic time point statistics based on animal model in Europe, Table S16: Chronic time point statistics based on animal model in Asia.
