# Supplementary material for: Intracortical Microelectrode Array Unit Yield under Chronic Conditions: A Comparative Evaluation
Source: Micromachines (Basel). 2021 Aug 17;12(8):972. doi: 10.3390/mi12080972 (PMC8400387; doi:10.3390/mi12080972)
Supplement: Supplementary file 1 [file micromachines-12-00972-s001.zip › micromachines-1249045-supplementary.pdf]

Supplementary Materials

Intracortical Microelectrode Array Unit Yield under Chronic Conditions: A Comparative Evaluation

Joshua O. Usoro [1], Brandon S. Sturgill [1], Kate C. Musselman [1], Jeffrey R. Capadona [2,3] and Joseph J. Pancrazio [1,*]

[1] Department of Bioengineering, The University of Texas at Dallas, Richardson, TX 75080, USA; joshua.usoro@utdallas.edu (J.O.U.); brandon.sturgill@utdallas.edu (B.S.S.); katecmusselman@gmail.com (K.C.M.)
[2] Department of Biomedical Engineering, Case Western Reserve University, Cleveland, OH 44106, USA; jrc35@case.edu
[3] Advanced Platform Technology Center, L. Stokes Cleveland VA Medical Center, Rehabilitation Research and Development, Cleveland, OH 44106, USA
* Correspondence: joseph.pancrazio@utdallas.edu

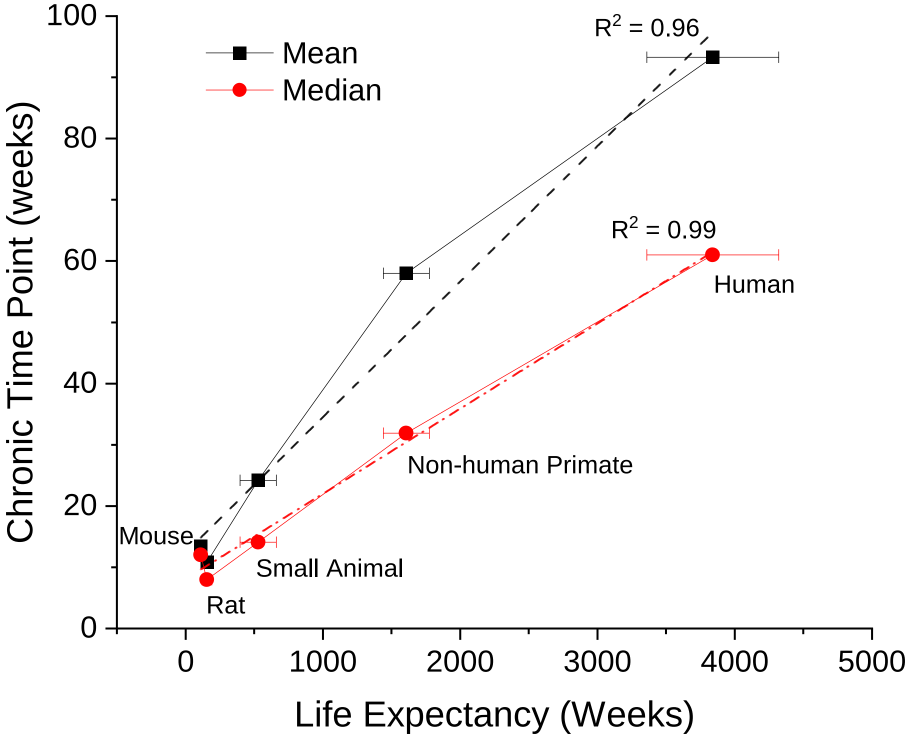

Figure S1. Chronic durations based on life expectancy. Mean (black square) and median (red circle) chronic duration are plotted as a function of the life expectancy of the animal model. Horizontal error bars reflect the average life span range of the animal model. Mean and median data points were fitted with a linear function indicated by the dashed lines. Adjusted R^2 values that indicate goodness of fit are reported as well.

Table S1. Chronic time point statistics based on device used

Metric (in Weeks)	Custom	Microwire	NeuroNexus	Blackrock
Number of Studies	71	23	25	43
Mean	19.1	23.8	9.98	59.5
Median	10.0	9.00	11.4	44.0
Range	0.429 - 34.7	2.00 - 156	0.429 – 27.0	2.00 - 282
Standard Deviation	29.1	34.1	6.74	57.8
References	[16,22,28,30–34,36,39,40,42,45–49,51–55,57,59–62,66,70–72,74,79,83,84,86,88–90,92–96,100,101,104,106–109,111,114,120,123,126–128,130,131,135,138,140,153,155,159,160,162,163,167]	[35,56,58,63,73,75,76,80–82,105,112,115,119,121,125,129,136,146,156,158,161,164]	[24–27,37,38,41,43,44,50,64,65,67–69,77,78,85,87,91,97,102,110,122]	[1,3,4,7,29,98,99,103,113,116–118,124,132–134,137,139,141–145,147–152,154,157,165,166,168–173,192]

Table S2. Chronic time point statistics based on device used in a mouse model

Metric (in Weeks)	Custom	Microwire	NeuroNexus	Blackrock
Number of Studies	13	1	8	0
Mean	13.9	8.00	14.4	N/A
Median	12.0	8.00	16.0	N/A
Range	1.00 – 34.7	N/A	0.429 – 27.0	N/A
Standard Deviation	9.90	N/A	7.33	N/A
References	[30–32,34,36,40,42,45,46,48,49,88,106]	[35]	[24,25,37,38,41,43,44,50]	N/A

Table S3. Chronic time point statistics based on device used in a rat model

Metric (in Weeks)	Custom	Microwire	NeuroNexus	Blackrock
Number of Studies	39	10	16	4
Mean	11.2	9.29	8.07	22.5
Median	8.00	5.50	6.00	24.0
Range	1.00–52.0	2.00–39.0	1.00–16.0	12.0–30.0
Standard Deviation	9.69	11.1	5.62	7.55

References	[16,22,28,33,48,51–55,57,59–62,66,70–72,74,79,83,84,86,89,90,92–96,100,101,104,106–109]	[56,58,63,73,75,76,80–82,105]	[26,27,38,64,65,67–69,77,78,85,87,91,97,102,110]	[29,98,99,103]

Table S4. Chronic time point statistics based on device used in a small animal model

Metric (in Weeks)	Custom	Microwire	NeuroNexus	Blackrock
Number of Studies	10	6	1	8
Mean	26.3	13.7	5.00	34.5
Median	17.2	6.86	5.00	39.5
Range	0.700–96.0	4.33–36.0	N/A	12.9–52.0
Standard Deviation	29.6	13.3	N/A	18.4
References	[111,114,120,123,126–128,130,131,135]	[112,115,119,121,125,129]	[122]	[113,116–118,124,132–134]

Table S5. Chronic time point statistics based on device used in a non-human primate model

Metric (in Weeks)	Custom	Microwire	NeuroNexus	Blackrock
Number of Studies	8	6	0	19
Mean	56.6	60.6	N/A	57.7
Median	23.0	37.6	N/A	44.0
Range	1.43–163	26.3–156	N/A	12.0–156
Standard Deviation	67.5	50.1	N/A	50.3
References	[138,140,153,155,159,160,162,163]	[136,146,156,158,161,164]	N/A	[3,124,133,137,139,141–145,147–152,154,157,192]

Table S6. Chronic time point statistics based on device used in a human model

Metric (in Weeks)	Custom	Microwire	NeuroNexus	Blackrock
Number of Studies	1	0	0	11
Mean	26.0	N/A	N/A	99.4
Median	26.0	N/A	N/A	70
Range	N/A	N/A	N/A	26.0–282
Standard Deviation	N/A	N/A	N/A	78.0
References	[167]	N/A	N/A	[1,4,7,165,166,168–173]

Table S7. Chronic time point statistics based on publication year

Metric (in Weeks)	≤ 2000	2001 - 2005	2006 - 2010	2011 - 2015	2016 - 2020
Number of Studies	14	13	29	48	56
Mean	35.2	25.6	25.1	34.5	26.9
Median	14.0	8.00	8.57	16.0	16.0
Range	3.71–163	1.00–156	1.00–156	1.00–282	0.429–172
Standard Deviation	52.1	43.9	32.0	50.9	35.0
References	[58,113,120–122,130–135,161–163]	[26,59–65,126,127,136,137,164]	[1,27,34,48,51,53,66–78,114,115,129,138–141,146,168]	[3,7,22,25,28,33,35–39,54–56,79–84,86–91,109,110,116,117,119,124,125,142–145,147–150,169–171,192]	[4,16,24,29–32,40–47,49,50,52,57,92–102,104–108,111,112,118,123,128,151–160,165–167,172,173]

Table S8. Chronic time point statistics based on publication year in a mouse model

Metric (in Weeks)	≤ 2000	2001 - 2005	2006 - 2010	2011 - 2015	2016 - 2020
Number of Studies	0	0	2	6	14
Mean	N/A	N/A	8.50	15.0	14.1
Median	N/A	N/A	8.50	16.0	12.0
Range	N/A	N/A	1.00–16.0	4.00–27.0	0.429–34.7
Standard Deviation	N/A	N/A	7.50	8.15	9.11
References	*N/A*	N/A	[48,72]	[25,35–39]	[24,30–32,40–47,49,50]

Table S9. Chronic time point statistics based on publication year in a rat model

Metric (in Weeks)	≤ 2000	2001 - 2005	2006 - 2010	2011 - 2015	2016 - 2020
Number of Studies	0	8	17	22	21
Mean	N/A	5.13	8.60	10.5	15.5
Median	N/A	5.00	6.00	7.00	13.0
Range	N/A	1.00–12.0	3.00–26.0	1.00–39.0	4.33–52.0
Standard Deviation	N/A	3.72	6.25	9.53	11.3

References	*N/A*	[26,59–65]	[26,27,48,51,53,66,67,69–78]	[22,28,33,38,54–56,79–91,109,110]	[16,29,98–107,52,108,57,92–97]

Table S10. Chronic time point statistics based on publication year in a small animal model

Metric (in Weeks)	≤ 2000	2001 - 2005	2006 - 2010	2011 - 2015	2016 - 2020
Number of Studies	10	2	3	5	5
Mean	15.7	23.7	33.2	31.9	32.7
Median	12.9	23.7	36.0	32.0	10.0
Range	3.71–52.0	21.4–26.0	4.33–59.3	4.33–52.0	0.700–96.0
Standard Deviation	14.0	3.23	27.6	19.1	40.9
References	[113,120–122,130–135]	[126,127]	[114,115,129]	[116,117,119,124,125]	[111,112,118,123,128]

Table S11. Chronic time point statistics based on publication year in a non-human primate model

Metric (in Weeks)	≤ 2000	2001 - 2005	2006 - 2010	2011 - 2015	2016 - 2020
Number of Studies	4	3	5	11	10
Mean	91.4	81.7	70.1	55.4	34.2
Median	93.9	76.0	52.0	52.0	25.0
Range	14.3–163	13.0–156	26.3–156	12.0–156	1.43–150
Standard Deviation	79.3	71.7	50.8	46.5	42.7
References	[133,161–163]	[136,137,164]	[138–141,146]	[3,124,142–145,147,149,150,192]	[152–160]

Table S12. Chronic time point statistics based on publication year in a human model

Metric (in Weeks)	≤ 2000	2001 - 2005	2006 - 2010	2011 - 2015	2016 - 2020
Number of Studies	0	0	2	4	6
Mean	N/A	N/A	35.5	142	80.1
Median	N/A	N/A	35.5	117	61.0
Range	N/A	N/A	32.0–39.0	52.0–282	26.0–172
Standard Deviation	N/A	N/A	3.50	101	60.2
References	*N/A*	N/A	[1,168]	[7,169–171]	[4,165–167,172,173]

Table S13. Chronic time point statistics based on geographical region

Metric (in Weeks)	North America	Europe	Asia	Other
Number of Studies	132	17	6	3
Mean	31.8	14.2	21.7	22.4
Median	14.3	12.0	7.00	30.3
Range	0.429–282	0.700–30.0	4.00–98.6	1.00–36.0
Standard Deviation	44.8	9.11	37.7	18.8
References	[1,4,7,16,22,24–30,32–44,58–109,112–118,120–124,126–128,130–145,147–156,161–166,168–173,192]	[31,48–50,53–57,111,119,125,146,157,159,160,167]	[3,45–47,51,52]	[110,129,158]

Table S14. Chronic time point statistics based on animal model in North America

Metric (in Weeks)	Mouse	Rat	Small Animal	Non-Human Primate	Human
Number of Studies	15	60	21	27	11
Mean	14.4	11.1	26.7	63.7	99.4
Median	16.0	8.00	14.3	44.0	70.0
Range	0.429–34.7	1.00–52.0	3.71–96.0	1.43–163	26.0–282
Standard Deviation	9.05	9.82	24.3	55.3	78.0
References	[24,25,30,32,35–44,72]	[16,22,26–29,33,38,58–109]	[112–118,120–124,126–128,130–135]	[124,133,136–145,147–156,161–164,192]	[1,4,7,165,166,168–173]

Table S15. Chronic time point statistics based on animal model in Europe

Metric (in Weeks)	Mouse	Rat	Small Animal	Non-Human Primate	Human
Number of Studies	3	6	3	4	1
Mean	19.3	11.7	9.68	16.1	26.0
Median	16.0	13.5	4.33	16.0	26.0
Range	12–30	3.86–17.3	0.700–24.0	6.00–26.3	N/A
Standard Deviation	9.45	5.57	12.5	10.5	N/A
References	[48–50]	[48,53–57]	[111,119,125]	[146,157,159,160]	[167]

Table S16. Chronic time point statistics based on animal model in Asia

Metric (in Weeks)	Mouse	Rat	Small Animal	Non-Human Primate	Human
Number of Studies	3	2	0	1	0

Mean	7.00	5.17	N/A	98.6	N/A
Median	8.00	5.17	N/A	98.6	N/A
Range	4.00–9.00	4.33–6.00	N/A	N/A	N/A
Standard Deviation	2.65	1.18	N/A	N/A	N/A
References	[45–47]	[51,52]	*N/A*	[3]	*N/A*

References

192. Callier, T.; Schluter, E.W.; Tabot, G.A.; Miller, L.E.; Tenore, F. V.; Bensmaia, S.J. Long-term stability of sensitivity to intracortical microstimulation of somatosensory cortex. *J. Neural Eng.* **2015**, *12*, doi:10.1088/1741-2560/12/5/056010.
